# Omeprazole does not Potentiate Acute Oxygen Toxicity in Fetal Human Pulmonary Microvascular Endothelial Cells Exposed to Hyperoxia

**DOI:** 10.4172/2153-2435.1000424

**Published:** 2015-10-09

**Authors:** Ananddeep Patel, Shaojie Zhang, Bhagavatula Moorthy, Binoy Shivanna

**Affiliations:** Section of Neonatology, Department of Pediatrics, Baylor College of Medicine, Houston, Texas, USA

**Keywords:** Omeprazole, Aryl hydrocarbon receptor, Hyperoxia, Oxidant stress, Human pulmonary microvascular endothelial cells, Cytochrome P450 1A1, NADP(H) quinone oxidoreductase 1

## Abstract

Hyperoxia contributes to the pathogenesis of broncho-pulmonary dysplasia (BPD), which is a developmental lung disease of premature infants that is characterized by an interruption of lung alveolar and pulmonary vascular development. Omeprazole (OM) is a proton pump inhibitor that is used to treat humans with gastric acid related disorders. Earlier we observed that OM-mediated aryl hydrocarbon receptor (AhR) activation attenuates acute hyperoxic lung injury in adult mice and oxygen toxicity in adult human lung cells. However, our later studies in newborn mice demonstrated that OM potentiates hyperoxia-induced developmental lung injury. Whether OM exerts a similar toxicity in primary human fetal lung cells is unknown. Hence, we tested the hypothesis that OM potentiates hyperoxia-induced cytotoxicity and ROS generation in the human fetal lung derived primary human pulmonary microvascular endothelial cells (HPMEC). OM activated AhR as evident by a dose-dependent increase in cytochrome P450 (CYP) 1A1 mRNA levels in OM-treated cells. Furthermore, OM at a concentration of 100 μM (OM 100) increased NADP(H) quinone oxidoreductase 1 (NQO1) expression. Surprisingly, hyperoxia decreased rather than increase the NQO1 protein levels in OM 100-treated cells. Exposure to hyperoxia increased cytotoxicity and hydrogen peroxide (H_2_O_2_) levels. Interestingly, OM 100-treated cells exposed to air had increased H_2_O_2_ levels. However, hyperoxia did not further augment H_2_O_2_ levels in OM 100-treated cells. Additionally, hyperoxia-mediated oxygen toxicity was similar in both vehicle- and OM-treated cells. These findings contradict our hypothesis and support the hypothesis that OM does not potentiate acute hyperoxic injury in HPMEC *in vitro*.

## Introduction

Although supplemental oxygen is commonly administered as a life-saving measure in patients with impaired lung function, it may also exacerbate lung injury [1]. Excessive oxygen exposure leads to increased reactive oxygen species (ROS) production and expression of pro-inflammatory cytokines [2], which can react with nearby macromolecules (e.g., protein, lipids, DNA, and RNA) and modify their structure and function [3], resulting in both acute and chronic pulmonary toxicities. The antioxidant defense system develops late in gestation, making preterm neonates highly susceptible to oxidative stress [4,5]. Despite significant advances in the management of premature neonates, broncho-pulmonary dysplasia (BPD) remains the most prevalent, and one of the most serious long-term sequelae of preterm birth, affecting approximately 14,000 preterm infants born each year in United States [6,7]. Hyperoxia-induced ROS generation is a major contributor to the development of BPD and its sequelae [8]. Infants with BPD are more likely to have long-term pulmonary problems, increased re-hospitalizations during the first year of life, and delayed neurodevelopment [6,9]. Hence, there is an urgent need for improved therapies in the prevention and treatment of BPD.

The aryl hydrocarbon receptor (AhR) is a member of basic - helix – loop – helix / PER – ARNT – SIM family of transcriptional regulators [10–12]. In humans, the AhR is highly expressed in the lungs, thymus, kidney and liver [13]. The AhR is predominantly cytosolic, held in a core complex comprising two molecules of 90-kDa heat shock protein and a single molecule of the co-chaperone hepatitis X-associated protein-2 [14,15]. AhR activation results in the translocation of the cytosolic AhR to the nucleus, where it dimerizes with the AhR nuclear translocator, to form a heterodimeric transcription factor. The heterodimeric transcription factor initiates transcription of many phase I and phase II detoxification enzymes such as cytochrome P450 (CYP) 1A1, CYP1A2, glutathione S-transferase-α, NAD(P)H quinone reductase-1 (NQO1), UDP glucuronosyl transferase which are encoded by the *Ah* gene locus [16–19]. Studies from our laboratory and elsewhere have demonstrated that AhR is a crucial regulator of lung inflammation and oxidative stress in adult and newborn mice [20–23]. Furthermore, we observed that AhR deficiency potentiates oxygen toxicity in primary fetal human pulmonary microvascular endothelial cells (HPMEC) [24]. These observations indicate that AhR activation may be sufficient to protect newborn mice and primary human lung cells against oxygen toxicity. Hence, we investigated whether an AhR agonist such as omeprazole (OM) can modulate hyperoxic lung injury in newborn animals and human lung cells.

Omeprazole, a substituted benzimidazole derivative, is a proton pump inhibitor (PPI) that inhibits gastric acid secretion in humans [25] and is used to treat gastric acid related disorders in humans [26]. OM is known to activate AhR in adult humans and rodents [27–32]. Hence, we investigated whether OM can activate AhR and attenuate hyperoxia-induced developmental lung injury in newborn mice. To our surprise, long-term OM therapy potentiated hyperoxic lung injury in newborn mice and these harmful effects was associated with decreased AhR activation [33]. Whether short-term OM therapy potentiates oxygen toxicity in primary human fetal lung cells is unknown. The goals of this study, therefore, were to investigate the effects of short-term OM therapy in fetal HPMEC exposed to acute hyperoxia. We specifically chose HPMEC because they are widely used to determine the mechanisms of neonatal lung injury [34,35]. Using these cells, we tested the hypothesis that OM potentiates acute hyperoxia-induced cytotoxicity and ROS generation in primary human fetal lung cells *in vitro*.

## Materials and Methods

### Cell culture and treatment

Human pulmonary microvascular endothelial cells (HPMEC), the primary microvascular endothelial cells derived from the lungs of human fetus were obtained from ScienCell research laboratories (San Diego, CA; 3000). HPMEC were grown in 95% air and 5% CO_2_ at 37°C in specific endothelial cell medium according to the manufacturer’s protocol. Cells were treated with either dimethylsulfoxide (DMSO) (Sigma-Aldrich, St. Louis, MO; 276855) or OM at varying concentrations up to 100 μM (Sigma-Aldrich, St. Louis, MO; O104) for 2 h, followed by exposure to air or hyperoxia for up to 48 h.

### Determination of functional activation of the AhR

It is widely established that functional activation of AhR results in its translocation into the nucleus, which results in transcriptional activation of the phase I enzyme, CYP1A1. Therefore, we determined the functional activation of AhR by analyzing the expression of CYP1A1 mRNA levels.

### Western blot assays

Whole-cell protein extracts from the cells treated with DMSO or OM and exposed to air or hyperoxia was obtained by using nuclear extraction kit (Active Motif, Carlsbad, CA; 40010) according to the manufacturer’s instructions [31]. β-actin was used as the reference protein. The protein extracts were separated by 10% SDS-polyacrylamide gel electrophoresis and transferred to polyvinylidene difluoride membranes. The membranes were incubated overnight at 4°C with the following primary antibodies: anti-NQO1 (Santa Cruz Biotechnologies, Santa Cruz, CA; sc-16464, dilution 1:500) and anti-β-actin (Santa Cruz Biotechnologies, Santa Cruz, CA; sc-47778, dilution 1:1000) antibodies. The primary antibodies were detected by incubation with the appropriate horseradish peroxidase-conjugated secondary antibodies. The immunoreactive bands were detected by chemiluminescence methods and the band density was analyzed by Image J software (National Institutes of Health, Bethesda, MD).

### Real-time RT-PCR assays

Cells were grown on 6 well plates to 50–60 % confluence, after which they were treated with DMSO or OM, and exposed to air for 24 h, following which the total RNA was isolated and reverse transcribed to cDNA as mentioned before [31]. Real-time quantitative RT-PCR analysis was performed with 7900HT Real-Time PCR System using iTaq Universal SYBR Green Supermix (Biorad, Hercules, CA; 1725121). The sequences of the primer pairs were *hCYP1A1*: 5′-TGGATGAGAAC-GCCAATGTC-3′ and 5′-TGGGTTGACCCATAGCTTCT-3′; *hNQO1*: 5′-ACGCCC-GAATTCAAATCCT-3′ and 5′-CCTGCCTGGAAGTT-TAGGTCAA-3′; *hβ-actin*: 5′-TGACGTGGACATCCGCAAAG-3′ and 5′-CTGGAAGGTGGACAGCGAGG-3′. β-actin was used as the reference gene. The ΔΔC_t_ method was used to calculate the fold change in mRNA expression: ΔC_t_ = C_t_ (target gene) − C_t_ (reference gene), ΔΔC_t_ = ΔC_t_ (treatment) − ΔC_t_ (control), fold change = 2^(−ΔΔCt)^.

### Exposure of cells to hyperoxia

Hyperoxia experiments were conducted in a plexiglass, sealed chamber into which a mixture of 95% O_2_ and 5% CO_2_ was circulated continuously. The chamber was placed in a Forma Scientific water-jacketed incubator at 37°C. Once the O_2_ level inside the chamber reached 95%, the cells were placed inside the chamber for the desired length of time.

### Cell viability assay

Cell viability was determined by a colorimetric assay based on the ability of viable cells to reduce the tetrazolium salt, MTT (3-(4, 5-dimethylthiazolyl-2)-2, 5-diphenyltetrazolium bromide), to formazan. HPMEC were seeded onto 96-well microplates, treated with DMSO or OM, followed by exposure to air or hyperoxia for up to 48 h. The cell viability was then assessed by MTT reduction assays as outlined in the MTT Assay protocol (American Type Culture Collection, Manassas, VA).

### Cell proliferation assay

Cell proliferation was determined based on the measurement of cellular DNA content via fluorescent dye binding using the CyQUANT NF cell proliferation assay kit (Invitrogen, Carlsbad, CA; C35006) as per the manufacturer’s recommendations. HPMEC seeded onto 96-well microplates were treated with DMSO or OM, followed by exposure to air or hyperoxia for up to 48 h. At the end of experiments, the medium was gently aspirated, and the cells were incubated for 30 minutes with 100 μl of 1X dye binding solution per well. Following the incubation, the fluorescence intensity of each sample was measured using Spectramax M3 fluorescence microplate reader with excitation at 485 nm and emission detection at 530 nm.

### Measurement of H_2_O_2_ generation

Hydrogen peroxide (H_2_O_2_) levels was quantified by the ROS-Glo^™^ H_2_O_2_ Assay (Promega, Madison, WI; G8820) according to the manufacturer’s recommendation. Briefly, cells were grown on 96-well plates to 60–70% confluence, after which they were treated with DMSO or OM, and exposed to air or hyperoxia for up to 24 h. Six-hours prior to the completion of experiments, the H_2_O_2_ substrate was added to the wells and the cell culture plates were returned to their respective exposure conditions for the final 6 h of the experiment. At the end of experiments, ROS-Glo^™^ detection solution was added to each well, following which the cells were incubated for 20 minutes at room temperature before the relative luminescence units was measured using Spectramax M3 luminescence microplate reader.

### Analyses of data

The results were analyzed by GraphPad Prism 5 software. At least three separate experiments were performed for each measurement, and the data are expressed as means ± SEM. The effects of OM and hyperoxia and their associated interactions for the outcome variables were assessed using ANOVA techniques. Multiple comparisons testing by the posthoc Bonferroni test was performed if statistical significance of either variable or interaction was noted by ANOVA. A *p* value of <0.05 was considered significant.

## Results

In this study, we investigated the impact of OM on acute hyperoxic injury in the human fetal lung derived HPMEC *in vitro*.

### Omeprazole increases CYP1A1 and NQO1 expression in HPMEC

**Figure 1:**
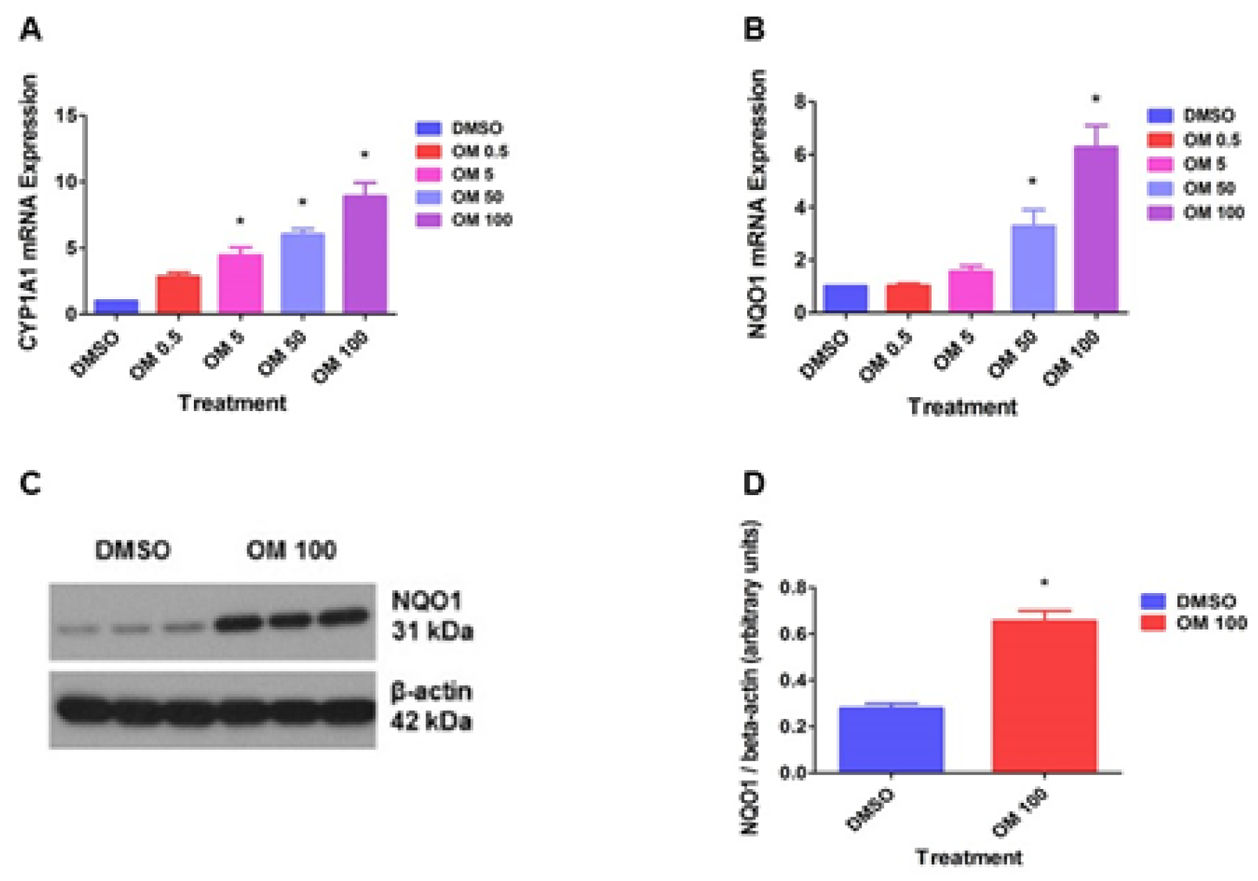
Omeprazole-treated HPMEC display increased CYP1A1 and NQO1 expression: Human pulmonary microvascular endothelial cells (HPMEC) were treated with dimethylsulfoxide (DMSO) or omeprazole (OM) at concentrations of 0.5 (OM 0.5), 5 (OM 5), 50 (OM 50) or 100 (OM 100) μM for up to 48 h, following which: RNA was extracted for CYP1A1 (A) and NQO1 (B) mRNA expression; and whole-cell protein was extracted for immunoblotting using anti-NQO1 or β-actin antibodies (C). Densitometric analyses wherein NQO1 band intensities were quantified and normalized to β-actin (D). Data are representative of at least three independent experiments. Values are presented as means ± SEM (n=3). *, p<0.05 vs. DMSO-treated cells.

It has been well documented that activation of AhR results in its translocation from the cytoplasm to the nucleus and results in transcriptional activation of the prototypical target gene, CYP1A1. Hence, we determined the effects of OM on CYP1A1 mRNA levels by real-time RT-PCR analysis. OM significantly increased CYP1A1 mRNA levels (Figure 1A) in a dose-dependent manner. We also determined the effects of OM on NQO1 expression since our earlier study indicated that this enzyme was down-regulated in AhR-deficient HPMEC [24]. Interestingly, OM increased NQO1 mRNA (Figure 1B) and protein levels (Figure 1C and 1D).

### Omeprazole does not potentiate hyperoxia-induced cytotoxicity in HPMEC

**Figure 2:**
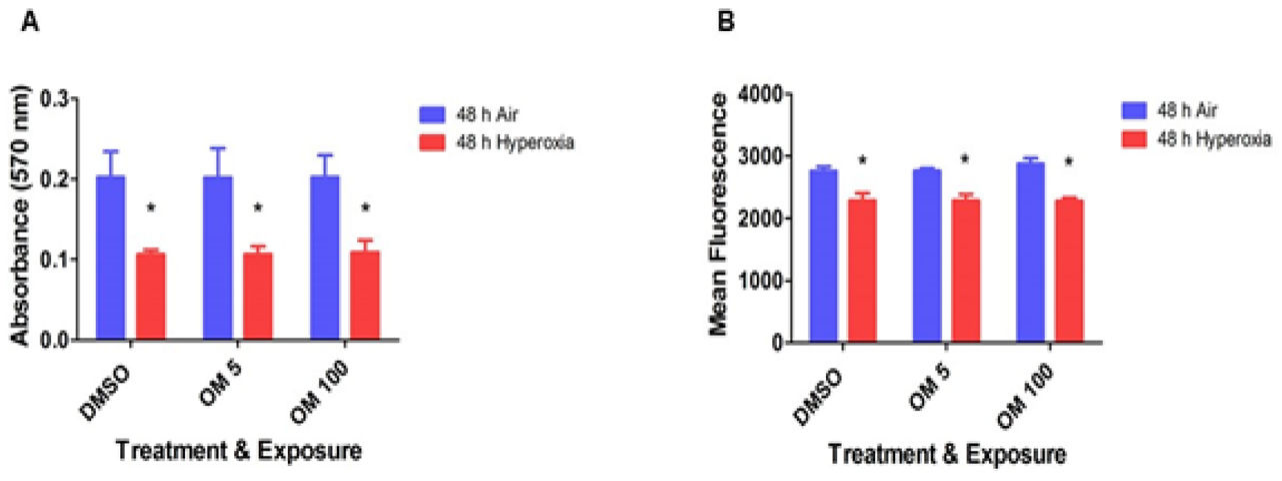
Effects of OM on hyperoxia-induced cytotoxicity in HPMEC: Human pulmonary microvascular endothelial cells (HPMEC) treated with DMSO, OM 5 or OM 100 were exposed to air or hyperoxia for up to 48 h, following which: (A) Cell viability was assessed by MTT (3-(4, 5-dimethylthiazolyl-2)-2, 5-diphenyltetrazolium bromide) reduction activities; and (B) Cell proliferation was determined based on the measurement of cellular DNA content via fluorescent dye binding using the CyQUANT NF cell proliferation assay. Data are representative of at least three independent experiments. Values are presented as means ± SEM (n=3). Two-way ANOVA showed an effect of hyperoxia but not of OM treatment for the dependent variables, cell viability and proliferation, in this figure. Significant differences between air- and hyperoxia-exposed cells are indicated by *, p<0.05.

The MTT activity reflects the mitochondrial activity of the cells, and thus the absorbance measured reflects the cell viability. Hyperoxia decreased cell viability, as reflected by a decline in the cellular capacities to reduce MTT (Figure 2A). Since oxidant-stress such as hyperoxia is well known to inhibit cell proliferation in general, we next determined its effects on cell proliferation. As expected, CyQUANT NF cell proliferation assay showed that hyperoxia inhibited proliferation of HPMEC (Figure 2B). However, OM did not potentiate hyperoxia-induced cytotoxicity (cell viability and proliferation) (Figure 2).

### Omeprazole does not increase hyperoxia-induced H_2_O_2_ generation in HPMEC

**Figure 3:**
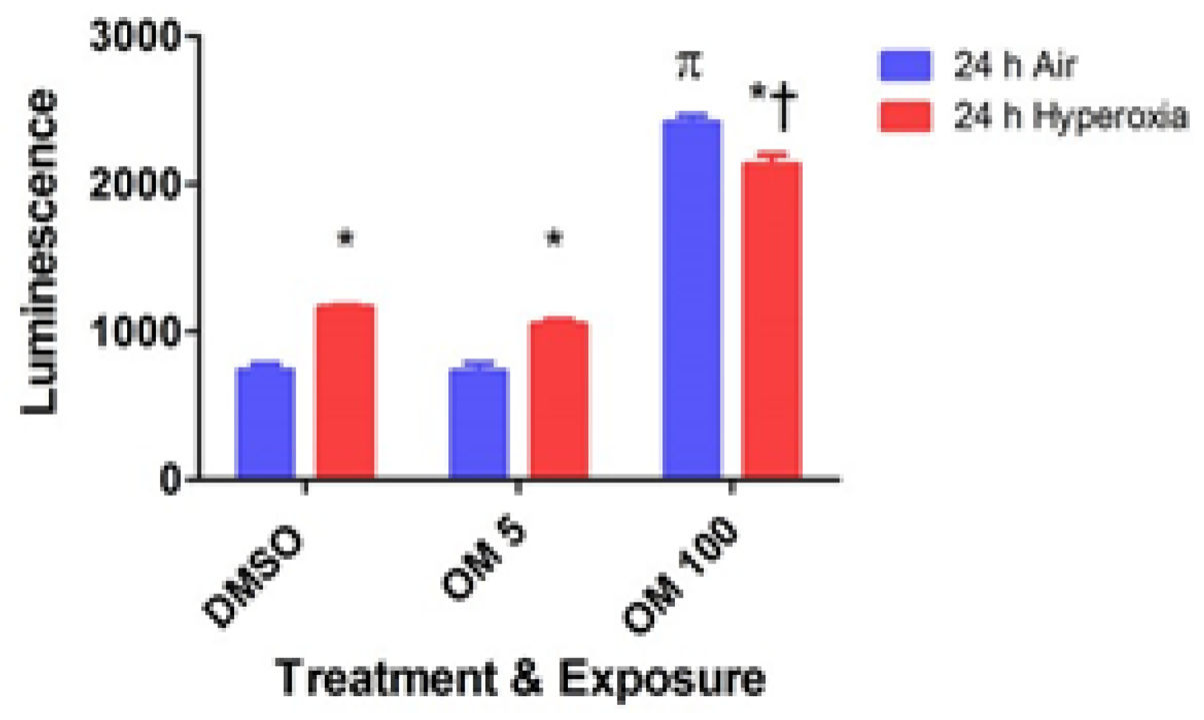
Effects of OM on hyperoxia-induced H_2_O_2_ generation in HPMEC: Human pulmonary microvascular endothelial cells (HPMEC) treated with DMSO, OM 5, or OM 100 were exposed to air or hyperoxia for up to 24 h, following which the H_2_O_2_ levels was measured by ROS-Glo^™^ H_2_O_2_ assay. Data are representative of at least three independent experiments. Values are presented as means ± SEM (n=3). Two-way ANOVA showed an effect of hyperoxia and OM 100 and an interaction between them for the dependent variable, H_2_O_2_ levels, in this figure. Significant differences between air- and hyperoxia-exposed cells are indicated by *, p<0.05. Significant differences between hyperoxia-exposed DMSO-, OM 5-, and OM 100–treated cells are indicated by †, p<0.05. Significant differences between air-exposed DMSO-, OM 5-, and OM 100–treated cells are indicated by π, p<0.05.

Hyperoxia-induced generation of ROS has been widely implicated in the pathogenesis of hyperoxic lung injury. To determine whether OM regulates ROS production, H_2_O_2_ levels was measured by ROS-Glo^™^ H_2_O_2_ assay. As expected, hyperoxia increased H_2_O_2_ levels in vehicle-and OM 5-treated cells (Figure 3). Interestingly, OM at a concentration of 100 μM increased H_2_O_2_ levels in HPMEC in air conditions (Figure 3). However, hyperoxia did not augment H_2_O_2_ levels in OM 100-treated cells and in fact, the H_2_O_2_ levels were decreased in hyperoxic-compared to air-conditions in OM 100-treated cells (Figure 3).

### Omeprazole does not augment hyperoxia-induced increase in NQO1 expression in HPMEC

**Figure 4:**
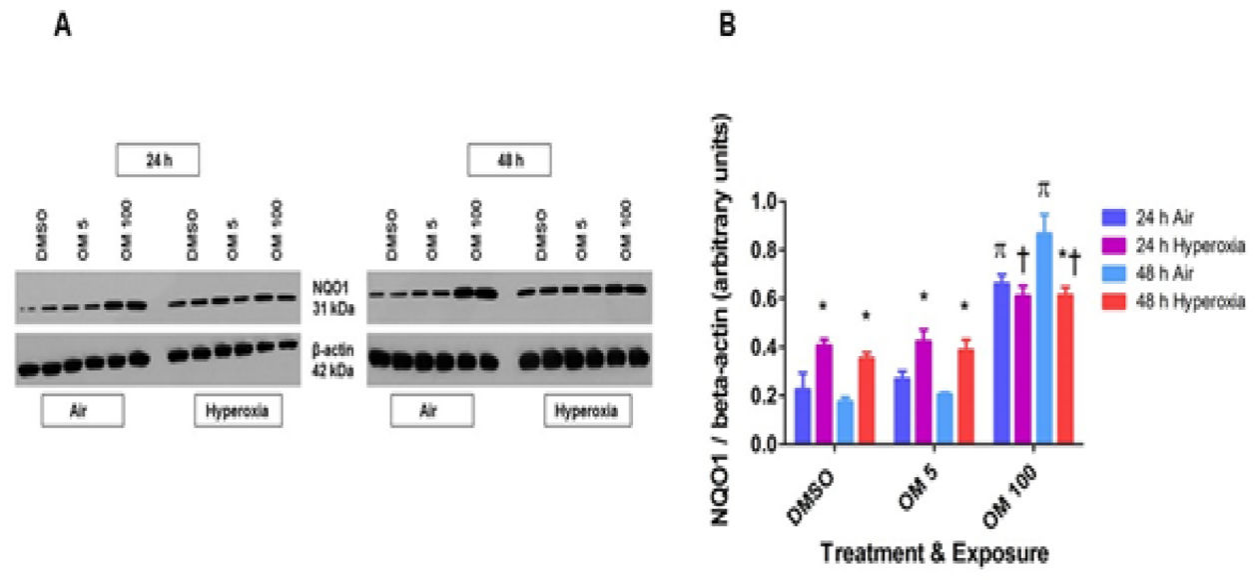
Effects of OM on NQO1 protein expression in hyperoxia-exposed HPMEC: Human pulmonary microvascular endothelial cells (HPMEC) were treated with DMSO, OM 5, or OM 100 for up to 48 h, following which whole-cell protein was extracted for immunoblotting using anti-NQO1 or β-actin antibodies (A). Densitometric analyses wherein NQO1 band intensities were quantified and normalized to β-actin (B). Values are presented as means ± SEM (n=3). Two-way ANOVA showed an effect of hyperoxia and OM 100 and an interaction between them for the dependent variable, NQO1 protein levels, in this figure. Significant differences between air- and hyperoxia-exposed cells are indicated by *, p<0.05. Significant differences between hyperoxia-exposed DMSO-, OM 5-, and OM 100–treated cells are indicated by †, p<0.05. Significant differences between air-exposed DMSO-, OM 5-, and OM 100–treated cells are indicated by π, p<0.05.

It is well established that phase I and II enzymes such as CYP1A1 and NQO1 attenuate hyperoxia-induced cytotoxicity. However, OM 100–treated cells failed to attenuate hyperoxia-induced cytotoxicity despite inducing the cytoprotective enzyme, NQO1, in HPMEC exposed to air. Hence, we determined the effects of OM on NQO1 protein expression in hyperoxic conditions. Hyperoxia increased NQO1 protein expression in vehicle- and OM 5-treated cells (Figure 4A and 4B). Consistent with its effect on H_2_O_2_ levels, hyperoxia decreased rather than increase the NQO1 protein levels in OM 100-treated cells (Figure 4A and 4B).

## Discussion

The present study demonstrates that the PPI, OM, does not potentiate acute hyperoxic injury *in vitro*. In human fetal lung-derived HPMEC *in vitro*, although OM 100 induced the cytoprotective enzyme, NQO1 in air conditions, it failed to augment hyperoxia-induced increase in NQO1 expression and protect against hyperoxia-induced cytotoxicity.

Studies from our laboratory and others have reported that AhR may be a crucial regulator of oxidant stress and inflammation through the induction of several detoxifying enzymes or via “cross-talk” with other signal transduction pathways and OM has shown to activate AhR. Additionally, we observed that OM attenuates hyperoxic injury in the adult human lung cell line, H441 cells *in vitro* [31]. However, we observed contrasting effects of OM on hyperoxia-induced lung pathology in newborn and adult mice [32,33]. Therefore, we conducted experiments with OM in human fetal lung derived HPMEC *in vitro*, to investigate whether OM modulates acute hyperoxic injury in primary human fetal lung cells.

Initially, we studied the interaction between OM and AhR. Functional activation of AhR results in its translocation into the nucleus, which causes transcriptional activation of the AhR gene battery that includes phase I and II detoxification enzymes such as CYP 1A1 and NQO1 [17–19,36]. Therefore, we analyzed the expression of the phase I and II enzymes to determine the functional activation of the AhR. The concentration-dependent increases in CYP1A1 and NQO1 mRNA expression in OM-treated cells exposed to air (Figure 1A and 1B), indicates that OM transcriptionally activates CYP1A1 and NQO1 enzymes. Upon further investigation of the concentration specific effects of OM on CYP1A1 and NQO1 mRNA expression (Figure 1A), we have observed that OM induces NQO1 via AhR-independent, but Nrf2-dependent mechanisms (manuscript under review).

Next, we studied the effects of OM on cell viability and cell proliferation in HPMEC exposed to hyperoxia. Evidence suggests that exposure to hyperoxia results in increased generation of ROS [37], and decreased cell proliferation [38,39] and viability [40]. Although the mechanism(s) are unclear, increased ROS levels have been thought to contribute to acute and chronic lung disease in humans by inhibiting cell proliferation and increasing cell death by apoptosis or necrosis [41]. Similarly, we observed increased H_2_O_2_ generation (Figure 3) and decreased cell viability (Figure 2A) and proliferation (Figure 2B) in hyperoxia-exposed HPMEC. However, OM did not augment hyperoxia-induced cytotoxicity (Figure 2), which supports the concept that short-term OM therapy does not potentiate acute oxygen toxicity in HPMEC. Importantly, higher OM concentrations increased H_2_O_2_ levels in air conditions (Figure 3). However, the H_2_O_2_ levels in fact decreased in OM 100-treated cells exposed to hyperoxia (Figure 3). The mechanisms of these interesting findings are unknown at this time point and warrants further investigation.

Our recent study in newborn mice suggest that OM therapy potentiates hyperoxia-induced developmental lung injury [33]. However our observations in this study disprove our hypothesis that OM potentiates acute hyperoxic injury in human fetal lung cells. The most plausible reason for these results may be related to the duration of OM therapy wherein we exposed human lung cells for a shorter duration (days) compared to the prolonged duration (weeks) in newborn mice. Some of the other contributory factors may be due to differences in the species and the cell type being studied.

To address the unexpected finding of the failure of OM to protect cells against hyperoxia-induced cytotoxicity despite inducing NQO1 in air conditions (Figure 1B, 1C and 1D), we determined the effects of OM therapy on NQO1 expression in hyperoxia-exposed cells. Surprisingly, hyperoxia decreased rather than the expected increase in NQO1 expression in OM 100-treated cells (Figure 4). Although the molecular mechanisms of these observations is beyond the scope of this study, it might be related to factors that may competitively inhibit the transcription of NQO1 gene at the promoter level or an altered miRNA profile that can inhibit the translation of NQO1 gene in OM 100-treated cells exposed to hyperoxia.

Hyperoxia-induced expression of the phase I and II enzymes have been observed by several other investigators both in adult [20,21,42,43] and newborn [44,45] rodents and human fetal lung cells [24]. This phenomenon might be a protective responsive to oxidative stress because of its striking resemblance to the effects of hyperoxia on the “classic” AOE such as superoxide dismutase, glutathione peroxidase, glutathione reductase, and catalase [46,47]. Furthermore, decreased expression of phase I and II enzymes in newborn AhRd mice and AhR-deficient human lung cells compared to wild type controls was associated with increased hyperoxic injury in our previous studies [23,24], which suggests that the effects of hyperoxia on these enzymes are a compensatory rather than a contributory response. NQO1 has been shown to protect cells and tissues against oxidant injury induced by various toxic chemicals [48] and oxygen [43,45,49]. The protective mechanisms of these enzymes have been attributed to their ability to conjugate and scavenge the reactive electrophiles and lipid peroxidation products generated by an oxidant injury [43,50]. The lack of OM 100-mediated protection against acute oxygen toxicity despite inducing NQO1 in air condition may be attributed at least in part due to its failure to increase the cytoprotective NQO1 enzyme levels in hyperoxic conditions (Figure 4).

In summary, we demonstrate that short-term OM therapy does not potentiate acute hyperoxia induced: (i) decrease in cell viability (Figure 2A); (ii) inhibition of cell proliferation (Figure 2B); and (iii) ROS generation (Figure 3) in HPMEC *in vitro*. Although, OM has a proven safety profile in humans for over decades and we did not observe any toxic effects with OM at concentrations similar to the blood concentrations (5 μM) documented in humans following OM therapy [51], it significantly increased H_2_O_2_ levels in air conditions (Figure 3) at a higher concentration (100 μM). Hence, we propose that there is a need to exert caution before instituting long-term high dose OM therapy in premature infants.

## Abbreviations

AhR: Aryl Hydrocarbon Receptor
ARDS: Acute Respiratory Distress Syndrome
BPD: Broncho Pulmonary Dysplasia
CYP: Cytochrome P450
DMSO: Dimethylsulfoxide
HPMEC: Human Pulmonary Microvascular Endothelial Cells
H_2_O_2_: Hydrogen Peroxide
MTT: 3-(4, 5-Dimethylthiazolyl-2)-2, 5-Diphenyltetrazolium Bromide
NQO1: NADP(H) Quinone Oxidoreductase 1
OM: Omeprazole
OM 5: Omeprazole 5 μM
OM 100: Omeprazole 100 μM
PPI: Proton Pump Inhibitor
ROS: Reactive Oxygen Species
